# Investigating the association between household exposure to *Anopheles stephensi* and malaria in Sudan and Ethiopia: A case-control study protocol

**DOI:** 10.1371/journal.pone.0309058

**Published:** 2024-09-03

**Authors:** Temesgen Ashine, Yehenew Asmamaw Ebstie, Rayyan Ibrahim, Adrienne Epstein, John Bradley, Mujahid Nouredayem, Mikiyas G. Michael, Amani Sidiahmed, Nigatu Negash, Abena Kochora, Jihad Eltaher Sulieman, Alison M. Reynolds, Eba Alemayehu, Endalew Zemene, Adane Eyasu, Alemayehu Dagne, Elifaged Hailemeskel, Fatou Jaiteh, Dereje Geleta, Ephrem Lejore, David Weetman, Ahmed Mahmoud Hussien, Fadwa Saad, Gudissa Assefa, Hiwot Solomon, Abdelgadir Bashir, Fekadu Massebo, Koen Peeters, Delenasaw Yewhalaw, Hmooda Toto Kafy, Martin J. Donnelly, Endalamaw Gadisa, Elfatih M. Malik, Anne L. Wilson

**Affiliations:** 1 Malaria and NTD Research Division, Armauer Hansen Research Institute, Addis Ababa, Ethiopia; 2 Department of Biology, College of Natural and Computational Sciences, Arba Minch University, Arba Minch, Ethiopia; 3 Department of Community Medicine, Faculty of Medicine, University of Khartoum, Khartoum, Sudan; 4 Department of Vector Biology, Liverpool School of Tropical Medicine, Liverpool, United Kingdom; 5 Department of Infectious Disease Epidemiology, London School of Hygiene and Tropical Medicine, London, United Kingdom; 6 Sennar Malaria Research and Training Centre (SMART Centre), Federal Ministry of Health, Khartoum, Sudan; 7 Tropical and Infectious Disease Research Centre, Jimma University, Jimma, Ethiopia; 8 Unit of Socio-Ecological Health Research, Department of Public Health, Institute of Tropical Medicine, Antwerpen, Belgium; 9 School of Public Health, College of Medicine and Health Sciences, Hawassa University, Hawassa, Ethiopia; 10 Primary Health Care General Directorate, Federal Ministry of Health, Khartoum, Sudan; 11 Disease Prevention and Control Directorate, Ethiopian Federal Ministry of Health, Addis Ababa, Ethiopia; 12 Directorate General of Global Health, Federal Ministry of Health, Khartoum, Sudan; Chiang Mai University Faculty of Agriculture, THAILAND

## Abstract

**Background:**

Endemic African malaria vectors are poorly adapted to typical urban ecologies. However, *Anopheles stephensi*, an urban malaria vector formerly confined to South Asia and the Persian Gulf, was recently detected in Africa and may change the epidemiology of malaria across the continent. Little is known about the public health implications of *An*. *stephensi* in Africa. This study is designed to assess the relative importance of household exposure to *An*. *stephensi* and endemic malaria vectors for malaria risk in urban Sudan and Ethiopia.

**Methods:**

Case-control studies will be conducted in 3 urban settings (2 in Sudan, 1 in Ethiopia) to assess the association between presence of *An*. *stephensi* in and around households and malaria. Cases, defined as individuals positive for *Plasmodium falciparum* and/or *P*. *vivax* by microscopy/rapid diagnostic test (RDT), and controls, defined as age-matched individuals negative for *P*. *falciparum* and/or *P*. *vivax* by microscopy/RDT, will be recruited from public health facilities. Both household surveys and entomological surveillance for adult and immature mosquitoes will be conducted at participant homes within 48 hours of enrolment. Adult and immature mosquitoes will be identified by polymerase chain reaction (PCR). Conditional logistic regression will be used to estimate the association between presence of *An*. *stephensi* and malaria status, adjusted for co-occurrence of other malaria vectors and participant gender.

**Conclusions:**

Findings from this study will provide evidence of the relative importance of *An*. *stephensi* for malaria burden in urban African settings, shedding light on the need for future intervention planning and policy development.

## Introduction

Africa currently has the highest rate of urbanization of any continent. The United Nations estimates that the world’s urban population will increase by 2.5 billion by 2050, with 90 percent of the growth in Asia and Africa [1]. Historically, the malaria burden in Africa has been concentrated in rural areas because African malaria vectors are not well adapted to urban ecologies [2–4]. However, *Anopheles stephensi*, a species of mosquito formerly confined to South Asia and the Persian Gulf but recently identified in Africa [5, 6], may change the epidemiology of malaria across the African continent. *An*. *stephensi* thrives in urban environments and is a highly competent vector for both *Plasmodium falciparum* and *P*. *vivax* [7, 8]; it therefore constitutes a potential new threat to African malaria control and hopes of elimination [5–7].

The WHO Malaria Threats Map highlights the current state of knowledge on *An*. *stephensi* detections across Africa with detections so far in Djibouti, Ethiopia, Sudan, Puntland, Nigeria, Somaliland, Ghana, Eritrea and Kenya [9]. Larval habitats of *An*. *stephensi* are typically man-made containers such as household/community water storage containers, construction water storage and overhead tanks, wells and drums, but it has also been identified in stream margins, sewage overflows, and flooded areas [10–12]. *An*. *stephensi* is an opportunistic vector, with biting behaviour driven by availability of hosts. Preliminary entomological surveillance in Ethiopia has revealed a propensity for resting in animal shelters [13, 14]. Surveillance indicates both indoor and outdoor biting, indoor and outdoor resting, and a preference for biting at dusk and during the night [15]. Further investigation is needed to determine if these behaviours are observed in African populations of *An*. *stephensi*. It is possible that additional vector control strategies will be necessary to control *An*. *stephensi*, in addition to current vector control tools that target vectors indoors (insecticide-treated nets [ITNs] and indoor residual spraying [IRS]).

There is a critical need to understand the public health impact of the threat posed by *An*. *stephensi*. An efficient urban malaria transmission cycle could turn cities and towns from areas with minimal malaria transmission to large-scale sources of infection, confounding global elimination efforts. *An*. *stephensi* was first detected in Djibouti in 2012 [5], where it was associated with a significant rise in malaria cases [7], from 1,684 malaria cases in 2013 to 72,332 confirmed cases reported in 2020 [16]. A recent dry season malaria outbreak in Dire Dawa, Eastern Ethiopia appears to be associated with *An*. *stephensi* [17]. Mathematical modelling also suggests that over 100 million people in cities across Africa are at risk of *An*. *stephensi* mediated malaria transmission [15]. Modelling by Hamlet *et al* suggests that annual *P*. *falciparum* malaria cases in Ethiopia could increase by 50% (95% CI 14–90) if no additional interventions are implemented [18]. Despite this growing evidence suggesting the potential involvement of *An*. *stephensi* in malaria transmission, there is great uncertainty about the malaria epidemiology in towns and cities in Sudan and Ethiopia; in particular, it is not known if, or to what extent, *An*. *stephensi* contributes to malaria transmission compared to native malaria vectors.

This manuscript describes a study protocol for a case-control study aimed at assessing the relative importance of *An*. *stephensi* and endemic malaria vectors for malaria burden in urban Sudan and Ethiopia. Case-control studies have been underused for malaria [19–23] but are well suited for our purpose since malaria cases are at present low in urban settings [24–26]. Results from this study will guide public health strategies for malaria control and elimination.

## Materials and methods

### Study design

We will conduct a community-based, age-matched case-control study in three study sites: two in Sudan and one in Ethiopia. Within these sites, cases and controls will be selected from health facilities over a 12-month period. Upon identification of cases and controls, entomological surveillance will be conducted at study participant households to assess the association between household entomological exposure and malaria case status.

### Study areas

Study sites have been selected using the following criteria: (1) presence of *An*. *stephensi*; (2) heterogeneity of *An*. *stephensi* density across a site; (3) ongoing malaria transmission; and (4) accessibility by study teams with minimal security threats. Entomological criteria (criteria #1 and #2) were assessed through ongoing entomological surveillance by study teams conducted at 61 sites in Sudan and 28 sites in Ethiopia. Epidemiological criteria (criterion #3) were assessed through using Health Management Information Systems (HMIS) data.

**Fig 1:**
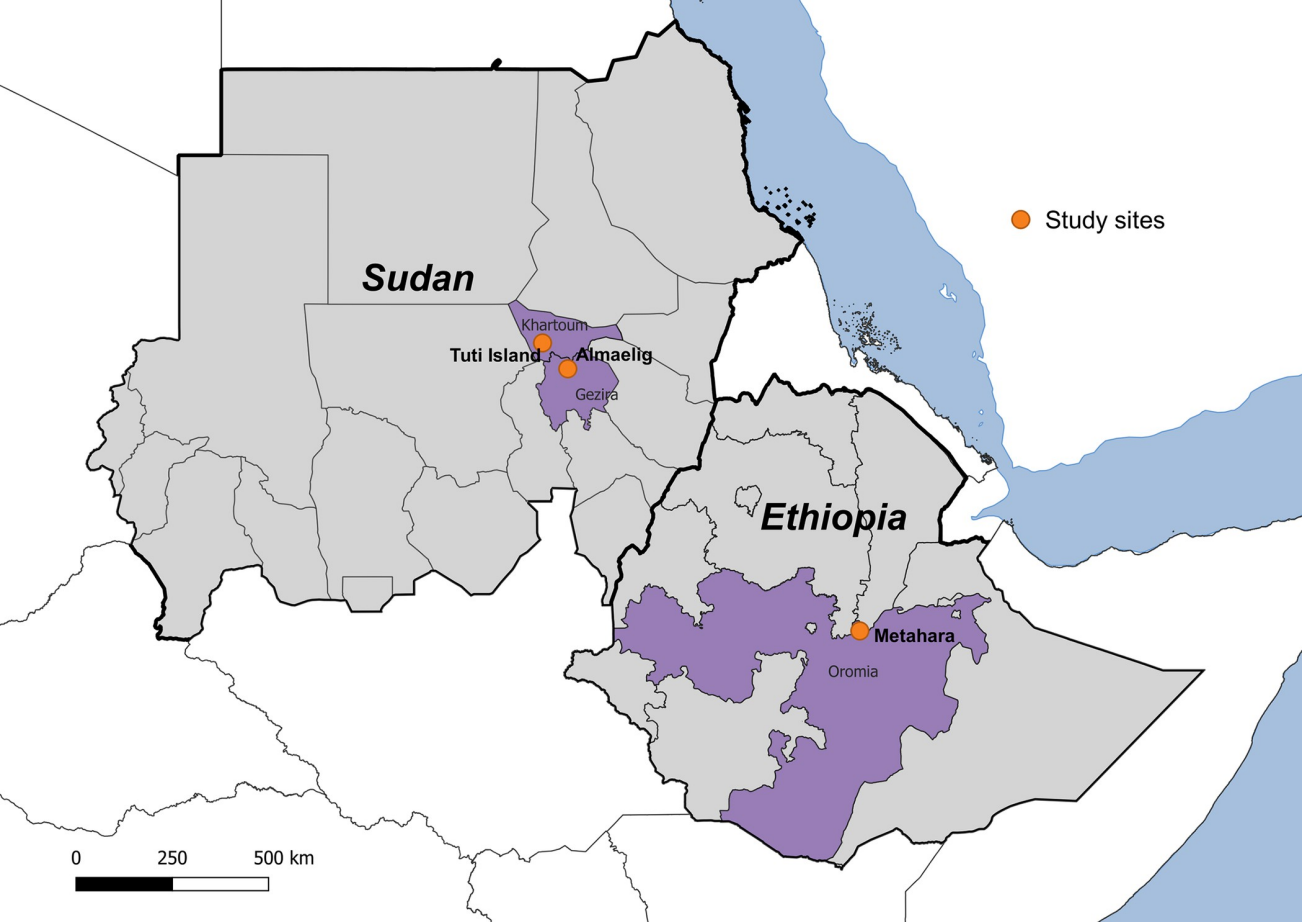
Location of case control study sites in Sudan and Ethiopia (Figure created from GADM data freely available for academic use and other non-commercial use, permitted under the CC BY 4.0 license).

In Sudan, the study will be conducted at two sites: Tuti Island in Khartoum State (15.621457, 32.504861) and Almaelig in Gezira State (15.017992, 33.094729) (Fig 1). *An*. *arabiensis* is considered the major malaria vector in both sites [27]. Tuti Island is an eight square kilometre island situated where the White Nile and Blue Nile meet in Sudan’s capital city, Khartoum, with a population of approximately 37,702. The island has a settlement, vegetable farms and orchards and is connected to the city via a single suspension bridge. *An*. *stephensi* was first detected in Tuti Island in 2018 [27] and a 2022 entomological survey found that 28% of randomly selected households had *An*. *stephensi* adults or larvae within 50 metres (Kafy *et al*, unpublished). Malaria transmission is seasonal, with a peak from October to December [28]. There is a single public health facility on Tuti Island from which cases will be recruited. The second site in Sudan, Almaelig, is a small town with a population of approximately 15,370. Almaelig is in the Gezira irrigation scheme which produces cotton, wheat, and groundnut. Presence of *An*. *stephensi* in Almaelig was first identified by our team in 2022 with 24% of randomly selected households positive for *An*. *stephensi* adults or larvae within 50 m of the home (Kafy *et al*, unpublished). Like Tuti, malaria transmission is seasonal with malaria transmitted by *An*. *arabiensis* (Kafy *et al*, unpublished). A total of four health facilities in Almaelig town and neighbouring settlements will be included for case and control selection: Almaelig Hospital, Aldibaiba, Alrayhana and Marakraka.

In Ethiopia, the study will be conducted in Metehara town, Oromia Region (8.903536, 39.917514) (Fig 1). Metehara town has a population of approximately 47,661. The main commercial activities are service-based and retail, and farming, including subsistence farming and a large government-owned sugar cane plantation, which supplies the government owned Metehara Sugar Factory located south of the town. Malaria transmission is seasonal with peaks from September to December. In Ethiopia, *An*. *arabiensis* is the main malaria vector while *An*. *pharoensis*, *An*. *funestus* and *An*. *nili* are secondary vectors [29]. Our team and others previously reported presence of *An*. *stephensi* in Metehara in 2022 with 18% of randomly selected households positive for either *An*. *stephensi* adults or larvae within 50m [30]. There are several public, faith-based, and private health facilities in the town. The study will recruit cases and controls at two public health centres, one serving Kebele 1 (Dire Gobu) and the other serving Kebele 2 (Haro Adi).

### Sample size

Entomological surveillance conducted in 2022 in and around 50 randomly selected households at each site informed the sample size calculations. Sampling for larval mosquitoes was conducted within 50m of each selected household. Similarly, adult collections were performed indoors and outdoors (within 50m of the household in Sudan, and within the compound in Ethiopia) using Prokopack aspirators and Centers for Disease Control miniature light traps (CDC LT). The proportion of households positive for *An*. *stephensi* larvae and/or adults was considered in the exposure probability for controls. A ratio of 1 case to 2 controls was adopted to increase statistical power given the identification of cases was thought to be the limiting factor in both settings.

In Sudan, across Tuti Island and Almaelig, 26% of households were exposed to *An*. *stephensi* adults or larvae (Kafy et al, unpublished). Assuming this (26%) exposure probability among controls, a 1:2 ratio of cases to controls, an odds ratio of 1.5, 20% correlation of exposure between cases and controls, 80% power, and a significance level of 5%, a total of 407 cases and 814 matched controls will be required. In Ethiopia, 18% of households in Metehara were exposed to *An*. *stephensi* adults or larvae (Ashine et al, unpublished). Assuming this (18%) exposure probability among controls, a 1:2 ratio of cases to controls, an odds ratio of 1.5, 20% correlation of exposure between cases and controls, 80% power, and a significance level of 5%, a total of 514 cases and 1028 matched controls will be required. Sample size calculations were run using the *power mcc* command in Stata (StataCorp. 2015. Stata Statistical Software: Release 14. College Station, TX: StataCorp LP.).

### Case and control definitions

The criteria for defining cases and controls are presented in Table 1. In both countries, the cases and controls or their caregivers must provide voluntary written informed consent to participate in the study, reside within the catchment of the health facility (within 30 minutes journey time) and be willing to be visited at home for additional data collection. In Ethiopia, an additional criterion will be that the participant must have been living in the study area for at least 4 weeks. This will restrict recruitment of non-locally derived cases given that there are seasonal workers who migrate to Metehara to work in the sugarcane plantation. The age range of cases and controls will differ between Sudan and Ethiopia. In Sudan, malaria cases are still predominantly in children; cases will therefore be aged greater than 6 months and less than 12 years. Controls will be matched to cases on two age groups: 6 months to less than 5 years; and 5 years and above to less than 12 years. In Ethiopia, HMIS data indicates that all ages are at risk for malaria; as such, cases and controls will be above 6 months of age with controls matched to cases on three age groups: above 6 months to less than 5 years; 5 years or above to less than 18 years; and 18 years and above. In Sudan, cases must be positive for *P*. *falciparum* and/or *P*. *vivax* detected using a rapid diagnostic test (RDT) (Bioline™ Malaria Ag P.f/P.v test), while controls must test negative by RDT. In Ethiopia, concerns over *pfhrp2/3* gene deletions [31] and lack of an appropriate and approved RDT means that cases must be positive for *P*. *falciparum* and/or *P*. *vivax* detected using microscopy, while controls must test negative by microscopy. Cases must have fever (axillary temperature ≥37.5°C) at the time of presentation or a history of fever within the previous 48 hours, while controls must be negative for fever (axillary temperature <37.5°C) or history of fever within the previous 48 hours. Cases and controls should have no signs or symptoms suggesting progression to severe malaria and should not have history of malaria treatment in the preceding two weeks. Controls must attend the same health centre as their matched case within 72 hours of case identification.

**Table 1:** Definition of cases and controls. Country specific criteria are indicated with acronyms, SUD for Sudan and ETH for Ethiopia.

Type	Case definition		Control definition	
Study criteria	Caregiver/participant gives informed consent to participate in the study		Caregiver/participant gives informed consent to participate in the study	
	Participant has been residing in the catchment area of the health facility (within 30 mins travel time) for at least 4 weeks		Participant has been residing in the catchment area of the health facility (within 30 mins travel time) for at least 4 weeks	
	Caregiver/participant willing to be visited at home for additional data collection		Caregiver/participant willing to be visited at home for additional data collection	
	SUD: Children aged above 6 months through 12 years	ETH: Individuals aged above 6 months	SUD: Children aged above 6 months through 12 years (matched to cases on age group, less than 5 years i.e. 6 months– 59 months, and more than 5 years i.e. 60 months– 144 months)	ETH: Individuals aged above 6 months (matched to cases on age group, 6 months– 5 years, 5–18 years, 18 years +)
	Available for household visit following visit to the health centre		Available for household visit following visit to the health centre	
Epidemiological criteria	Fever (axillary temperature ≥37.5°C) or history of fever in previous 48 h		Negative for fever (axillary temperature <37.5°C) or history of fever within the previous 48 h	
	SUD: Positive RDT for either *P*. *falciparum* or *P*. *vivax*	ETH: Positive microscopy for either *P*. *falciparum* or *P*. *vivax*	SUD: Negative RDT for *P*. *falciparum* and *P*. *vivax*	ETH: Negative microscopy for *P*. *falciparum* and *P*. *vivax*
	No signs or symptoms suggesting progression to severe malaria		No signs or symptoms suggesting progression to severe malaria	
	No history of malaria treatment in the preceding two weeks		No history of malaria treatment in the preceding two weeks	
	Attending health centre as outpatient (not admitted) within 72 h of control		Attending same health centre as outpatient (not admitted) within 72 h of case	

### Study procedures

#### Identification and enrolment of study participants and blood sample collection

At each health facility, staff of the health facility trained by the project teams will be responsible for screening and enrolment in collaboration with the rest of the health facility staff. Potential study participants attending the health centre as outpatients, or their caregivers will be approached and invited to participate in the study. The individual or their caregiver will be provided with information about the study, have an opportunity to ask questions, and will be asked to consider providing written informed consent to participate in the study. Children aged 8 years and above in Sudan and aged 11–17 years in Ethiopia will be asked to provide verbal assent to participate in the study. If the child does not assent, then they will not be included.

In both Sudan and Ethiopia, recruitment will be integrated with standard care as much as possible with RDTs and microscopy performed by health facility laboratory staff. A finger-prick blood sample will be taken to perform an RDT (Bioline™ Malaria Ag P.f/P.v test) in Sudan or microscopy in Ethiopia where thick and thin blood films will be prepared and stained with Giemsa. In addition, dried blood spots will be collected using filter paper to be analysed for the presence of 18s rRNA gene using PCR [32, 33]. Dried blood spots will also be stored for future molecular analyses to determine the proportion of *P*. *vivax* cases that are relapses and for whole genome sequencing of all species of *Plasmodium* [34, 35].

#### Household survey

Cases and controls will be visited at home by study personnel within 48 hours of enrolment.

Study fieldworkers will conduct a household survey to collect information on several variables (Table 2), including demographics, house structure, use of malaria preventive methods, and environment. Socio-economic status will be assessed using an asset index from an established questionnaire [36]. The condition of ITNs will be assessed and a proportionate hole index calculated according to established methods [37]. Households will also be mapped using a GPS receiver.

**Table 2:** Variables to be assessed during the household survey.

Socio-demographic	Gender
	Education (for children aged 7 and up)
	Caregiver education level
	Caregiver occupation
	Socio-economic position
Housing	Roof type
	Presence of ceiling
	Wall type
	Floor type
	Eave status of the sleeping space (closed, partially open, open)
	Presence and condition of any window screening
	Household size
	Number of sleepers in same room
	Functioning fan in the sleeping room
Personal protection	Time to bed and time to rise
	Typical outdoor activities in evening and early morning
	Typical sleeping location of the participant (indoor/outdoor)
	Reported ITN ownership
	Reported ITN use during the previous night
	Brand of ITN
	Number of ITNs in the sleeping space
	Age of ITN
	Condition of ITN (proportionate hole index)
	Number of adults or children sharing the same ITN as the case/control
	Walls sprayed with insecticide (IRS) in previous 12 months
	Use of other personal protection methods (coils, sprays etc)
	Elevation
Environment	Presence of animals in sleeping space or within 10 m
	Travel history in previous 2 weeks
	Location of any travel in previous 2 weeks
	Presence of another individual in the same household with fever or signs of malaria in past 2 weeks

#### Entomological survey

During the household visit, fieldworkers will conduct entomological surveillance for both adults and immature mosquitoes both inside and outside the participant household (within 50 m). Adult mosquito surveillance will be performed both indoor and outdoor using CDC LTs and Prokopack aspirators (both John W. Hock Company, Florida, USA). CDC LTs will be placed indoors at the foot end of an occupied bed net 1.5m off the floor and collections will be conducted for a 12-hour period overnight. Prokopack aspiration will be conducted inside and outside of the house between 5.00–6.00 AM before the CDC LT is retrieved. Collections will be performed for 20 minutes, with an additional 10 minutes for any out-buildings sheltering animals. Prokopack aspiration will also be performed in potential outdoor resting sites such as house exteriors, grain or rice stores, wood stacks, water pipes, drains, wells, trees, and bushes. In addition, Biogents Pro CDC-style traps (Biogents AG, Regensburg, Germany) will be deployed outdoor. BG Pro traps with lure will be placed outside the main dwelling structure close to presumed mosquito resting places, suspended 1.5m off the floor, and out of direct sunlight, wind, or heavy rain. BG Pro traps will be deployed with BG Lures releasing artificial human skin odour (Biogents AG, Regensburg, Germany) and light and run for 12 hours overnight. Immature collections will be done by systematically sampling aquatic habitats within 50 m of the study participant house using a standard dipper [38]. Both adult and larval collections will be done on one occasion only.

#### Additional entomological surveillance

We will attempt to determine whether collections of larvae and adult anophelines in and around the homes of cases and controls shortly after recruitment can be used to infer the presence of larvae/adult and mosquito density at the time of malaria transmission, approximately 10 days previously. Here we will adopt a similar approach to a previous case-control study conducted in The Gambia [19]. Entomological surveillance of larvae and adult anophelines will be performed within 48 hours of recruitment and repeated 10 days later for 20% of all cases and controls. The presence/absence of larvae/adult and the density of *An*. *stephensi* and other anopheline species will be compared between the two catches in the same location for quality assurance.

Due to low catch numbers of adult *An*. *stephensi* in Ethiopia, additional entomological surveillance will be conducted every two months. An initial census conducted in December 2023 suggests the presence of permissive and *An*. *stephensi* positive aquatic habitats across Metehara. The two Kebeles (Dire Gobu and Haro Adi) will be divided into 8 quadrants (4 quadrants per Kebele) and sampling efforts allocated to each proportional to household and potential aquatic habitat density. Every 2 months, permissive aquatic habitats identified in the census will be surveyed for *An*. *stephensi*, along with a subset of habitats selected purposively during each sampling round. Human and animal structures within 20 metres of each aquatic habitat will be surveyed for adult *An*. *stephensi* using Prokopack aspiration, CDC LT and BG Pro traps.

#### Mosquito species identification

For adult collections, mosquitoes will be sorted to anophelines and culicines. Anophelines will be identified morphologically [39]. Morphological identification of adults and larvae will be confirmed using polymerase chain reaction (PCR) [40]. To reduce the number of PCRs run, larvae will be pooled into groups of 20 samples according to their catch location.

#### Blood meal source and infection rate determination

Blood meal analysis of adults will be conducted; abdomens of freshly blood-fed *An*. *stephensi* and other malaria vectors will be subjected to amplification [41]. Furthermore, adult *An*. *stephensi* and a random sample of other malaria vectors will be screened for *P*. *falciparum* and *P*. *vivax* DNA. qPCR amplification will target the SSU RNA gene with species-specific primers [32].

### Data management

Study data will be collected and managed using REDCap [42, 43] electronic data capture tools hosted at the Liverpool School of Tropical Medicine (for Sudan) and Armauer Hansen Research Institute (for Ethiopia).

### Statistical analysis plan

#### Primary

The primary analysis for this study will include three separate exposure variables: 1) presence/absence of adult *An*. *stephensi* in and around the household; 2) presence/absence of immature *An*. *stephensi* in and around the household; and 3) combined presence/absence of adults and/or immature *An*. *stephensi* in and around the household. Should sufficient *An*. *stephensi* be caught, additional analyses will assess the impact of adult vector density on malaria status. Association between exposure variables and case status will be assessed using conditional logistic regression. Covariates will include co-occurrence of other anopheline vectors and participant gender. Effect estimates will be expressed as odds ratios with 95% confidence intervals.

#### Secondary

A similar approach to the above will be used to assess the associations between other key risk factors for malaria and malaria case status. These include (but are not limited to) presence/absence and density of adult and/or immature endemic malaria vectors, ITN use, ITN quality, travel history, and proximity of the sleeping space to animals. The distance between case and control households and *An*. *stephensi* positive habitats or structures identified in the overlaid entomological surveillance will be assessed, taking into account the most recent surveillance round only.

#### Sensitivity analysis

While the primary analysis will include all cases and controls recruited into the study, a sensitivity analysis will be conducted to adjust for potential misclassification of cases and controls. Cases with negative PCR for *Plasmodium* species will be excluded, as will controls with positive PCR results.

### Community sensitisation

Meetings have been held to introduce the study to relevant stakeholders in each country, including the Federal Ministry of Health, Regional Health Bureau, and City Administration Health Office in Ethiopia and Federal and State Ministry of Health, Locality Health Affairs, and the facility director in Sudan. During these meetings we presented the objectives of the research, research activities and potential implications for policy. Furthermore, regional health bureaus from the selected sites have been informed of the research and the methods involved. Prior to the start of the study, we will hold meetings with community leaders in the study sites (e.g. urban dwellers association in Ethiopia, Development & Public Services committees, and Health Facility Development Committee in Sudan) to inform them about the project and give them an opportunity to ask questions.

### Ethics

This study received ethical approval from the Liverpool School of Tropical Medicine Research Ethics Committee (Ref # 22–005, Ethiopia: 27 Jan 2023, Sudan: 18 Aug 2022), the London School of Hygiene and Tropical Medicine Research Ethics Committee (Ref # 28287, 24 Nov 2022), the Republic of Sudan National Health Research Ethics Review Committee (Ref # 8-1-21, 7 Feb 2021), the Armauer Hansen Research Institute Ethics Review Committee (Ref # PO-35-22, 20 Aug 2022), and the National Ethics Review Committee in Ethiopia (Ref # 1724642123, 23 Jan 2023).

We will adhere to guidelines set forth by the Declaration of Helsinki. All staff and investigators will receive training on human subjects protection, safeguarding and study-specific standard operating procedures (SOPs). Data collection activities (both epidemiological and entomological) will require written informed consent from the study participant or their caregiver. Consent forms are translated into local languages and explain in-depth the purpose of the study, procedures, risks and benefits, and the voluntary nature of participation.

### Dissemination

Findings will be disseminated in the study health facilities and communities through meetings to which local community members and local stakeholders will be invited. During these meetings, plain language summaries of the key findings of the study will be presented. Findings will be presented through meetings and briefs to stakeholders including the Federal Ministry of Health and National Malaria Control Programme. Internationally, findings from this work will be presented through peer-reviewed publications and presentations at scientific conferences and international policy fora.

## Discussion

This case-control study will provide evidence of the relative impact of *An*. *stephensi* on malaria in Ethiopian and Sudanese urban settings as compared to endemic *Anopheles* species. Recruiting cases and controls from health facilities and conducting entomological surveillance in and around houses will determine whether household-level exposure to *An*. *stephensi* is associated with greater risk of malaria. This study employs a unique design, pairing epidemiological principles with entomological surveillance.

To date, little is known about the impact of *An*. *stephensi* on malaria burden in urban Africa, but some preliminary evidence points to a potential positive association. A 2019 paper from Djibouti describes a simultaneous rise in *An*. *stephensi* occurrence and malaria cases from 2013–2017, including an increase in *P*. *vivax* cases [7]. While not able to draw causal conclusions, this paper provides descriptive evidence for the potential that the rise in *An*. *stephensi* contributed to malaria transmission. To support this finding, the authors detected a 3.1% sporozoite rate among captured adult *An*. *stephensi* females. Furthermore, a case-control study conducted during a dry season malaria outbreak in 2022 in Dire Dawa, Eastern Ethiopia found that presence of *An*. *stephensi* adults and/or larvae was associated with 3.30-times the odds of malaria cases compared to controls (95% CI 1.65–6.47) [17]. Importantly, abundance of *An*. *stephensi* in this setting was markedly high: all *Anopheles* larvae collected from artificial containers were identified as *An*. *stephensi*, and 97% of adult *Anopheles* mosquitoes were *An*. *stephensi*. The case-control study proposed here will further contribute to this body of evidence, expanding both geographically and temporally, and allowing for the assessment of potential seasonal changes in the associations between *An*. *stephensi* and malaria risk through the 12-month study period. This study will also include important urban settings, including those that rely heavily on irrigation schemes and plantations.

This study design is not without challenges and limitations. Firstly, the design assumes that entomological exposures around households are relatively stable. To test this assumption, entomological surveillance will be repeated after 10 days for a subset of all cases and controls. If the correlation between entomological collections is low, findings from this study may be attenuated. Secondly, this study will assess household-level exposure to vectors, yet malaria transmission could take place outside of the household, in the workplace or during travel. To better understand this, household surveys will collect information on employment, school attendance, and travel history. Finally, from an operational perspective, harmonizing the study in two countries with distinct settings and malaria profiles poses challenges. Substantial work has been done to tailor the studies to each setting, while maintaining the core design across the two countries. Civil unrest in Sudan beginning in April 2023 presents further challenges to the implementation of the study; for this reason, protocol details, including study locations, are subject to change. Civil unrest in Ethiopia close to Metehara may also hinder study progress.

This study will address the critical need to understand the public health impact of the threat posed by *An*. *stephensi*. With this understanding, future work can evaluate how existing control interventions can be used against the vector and develop additional strategies to protect urban populations from malaria.

## Abbreviations

CDC LT: CDC light trap
CI: confidence interval
ETH: Ethiopia
IRS: indoor residual spraying
ITN: insecticide-treated net
M: meter
PCR: polymerase chain reaction
SOP: standard operating procedure
SUD: Sudan
RDT: rapid diagnostic test
